# Silver spoons, reproduction, and growth catch-up in eastern Grey kangaroos

**DOI:** 10.1093/beheco/araf017

**Published:** 2025-02-26

**Authors:** Kelly Forrester, Andrew M MacDonald, Marco Festa-Bianchet

**Affiliations:** Département de Biologie, Université de Sherbrooke, Sherbrooke, 2500 Boulevard de l’Université, Sherbrooke, QC J1K 2R1, Canada; Département de Biologie, Université de Sherbrooke, Sherbrooke, 2500 Boulevard de l’Université, Sherbrooke, QC J1K 2R1, Canada; Département de Biologie, Université de Sherbrooke, Sherbrooke, 2500 Boulevard de l’Université, Sherbrooke, QC J1K 2R1, Canada; Research School of Biology, The Australian National University, 134 Linnaeus Way, Acton, ACT 2601, Australia

**Keywords:** catch-up growth, early life growth, early reproduction, growth reproduction trade-offs, indeterminate growth, life history theory, silver spoon effects, von Bertalanffy growth function

## Abstract

Early growth and environmental conditions can shape life-history trajectories. Long-lived iteroparous species with indeterminate growth face growth-reproduction trade-offs for most of their lives. Poor early conditions can delay primiparity and restrict growth, potentially compensated for by faster growth later in life, at the cost of reduced reproduction. We explored the variation in age at primiparity and early reproduction in eastern Grey kangaroos, based upon 13 yr of monitoring about 100 known-age females. We then examined associations between early reproduction, later reproduction, and lifetime growth. We used a modified von Bertalanffy growth function to model the indeterminate growth of females and to test the effects of early reproduction on lifetime growth. Favorable environmental conditions, large size, and condition as a subadult led to earlier reproduction and higher reproductive success at ages 3 to 5. As females aged, size and condition had diminishing effects on reproductive success. Females with greater early-adulthood reproduction had slightly higher reproduction later in life. We did not detect a growth cost of early reproduction. Large females in good condition favored early reproduction over growth, while those with poor early growth exhibited growth catch-up at the cost of reproduction both early and later in life. As reported for other long-lived iteroparous species with indeterminate growth, female kangaroos prioritize growth over reproduction for much of their lifespan. Eastern Gray kangaroos show heterogeneity in early growth and reproductive strategies. Early primiparity and reproduction are constrained by body condition, size, and environmental conditions when females are pre-reproductive subadults.

## Introduction

Early growth is a foundational phase of life-history trajectories, with potential lifelong effects. When resource are limited, natural selection favors resource allocation that maximize fitness through trade-offs in life-history traits, resulting in diverse life-history strategies and fitness heterogeneity (Stearns 1989; Roff and Fairbairn 2007; Wilson et al. 2011). Growth, reproduction, and maintenance trade-offs are well documented. Environmental conditions, maternal care, resource acquisition and allocation, and reproduction can affect growth (Harrison and Clegg 1969; van Noordwijk and De Jong 1986; Kunz and Hood 2000; Therrien et al. 2007; Gélin et al. 2016b; Pontzer and McGrosky 2022).

Life histories can be classified along a continuum from fast, prioritizing reproduction over survival, to slow, prioritizing survival over reproduction, but this continuum is not necessarily reflected in individuals within populations (Reynolds 2003; Stearns and Rodrigues 2020; Van de Walle et al. 2023). In stochastic environments, natural selection favors iteroparity to navigate resource fluctuations by allocating energy strategically to reproduction across multiple years (Varpe and Ejsmond 2018). Flexibility in resource allocation by long-lived iteroparous species can diversify lifelong growth patterns (Sebens 1987; Jokela 1997; Heino and Kaitala 1999).

The fitness advantages of large size can be limited by growth costs (Stoks et al. 2005; Rose et al. 2009; Dmitriew 2011; Clarke 2019). Growth trajectories can be influenced by genetics, resource availability, physiological limitations, environmental conditions, predation, and maternal care (Efstratiadis 1998; Van Buskirk and Yurewicz 1998; Johnston et al. 2003; Festa-Bianchet et al. 2004; Karkach 2006; Christiansen et al. 2018; Westrick et al. 2020). Vertebrates with determinate growth reach asymptotic skeletal size at or soon after sexual maturity (Sebens 1987; Lui and Baron 2011). Species with indeterminate growth continue to grow after sexual maturation, and in some cases over the lifetime, with high adult size plasticity, largely shaped by environmental conditions (Sebens 1987; Heino and Kaitala 1999; Vrtílek and Reichard 2015). Species with indeterminate growth face lifelong growth-reproduction trade-offs as larger size implies higher future reproductive success (Stearns 1992; Festa-Bianchet et al. 1998; Pomeroy et al. 1999). Individuals with poor initial growth can exhibit compensatory or catch-up growth, which often implies fitness costs (Nicieza and Metcalfe 1997; Metcalfe and Monaghan 2001).

Favorable early-life conditions, termed silver-spoon effects, can be associated with earlier primiparity, and enhanced reproductive success, survival, and growth (Grafen 1988; McAdam and Boutin 2003; Descamps et al. 2008; Hastings et al. 2011; Martin and Festa-Bianchet 2012; Wightman et al. 2022). Age of primiparity increases with high density and decreased food availability (Jorgenson et al. 1993; Wikenros et al. 2021). Similar adverse conditions can negatively affect life-history trajectories, reducing longevity and reproduction (Pigeon et al. 2017). Shared early conditions, or cohort effects, can have lifelong effects, but their impact often diminishes with age in large herbivores (Douhard et al. 2013; Herfindal et al. 2015; Hamel et al. 2016). Earlier reproduction in iteroparous species can be associated with greater lifetime reproductive success, decrease survival and longevity, or have no detectable impacts (Reiter and Le Boeuf 1991; Descamps et al. 2006; Martin and Festa-Bianchet 2012; Allain et al. 2023). Early-life environmental variance can explain a substantial portion of longevity and adult mass variation in large herbivores (Hamel et al. 2009).

Female eastern Gray kangaroos (*Macropus giganteus*) are large, long-lived, iteroparous, herbivorous marsupials that fit Karkach’s (2006) definition of indeterminate growth, growing many years after sexual maturity (Figure S1; Poole 1982). Larger female kangaroos are more fecund, but reproduction reduces subsequent growth (Gélin et al. 2015; Quesnel et al. 2018; Toni et al. 2020). Marsupials have short gestation and prolonged lactation, and thus can easily terminate allocation to offspring when resources are scarce, preferentially allocating to maintenance over reproduction (Kirsch 1977; Parker 1997; Low 1978; Thompson and Nicoll 1986; Toni et al. 2020). At each reproductive opportunity, female kangaroos face a trade-off between resource allocation to growth or reproduction, especially in early adulthood, making them useful models for studying life history trade-offs (Quesnel et al. 2018). To our knowledge, no studies have investigated how early-life environmental conditions and reproduction affect skeletal growth over multiple years after sexual maturity in mammals.

Here, we first explore how early-life morphological traits and environmental conditions affect age at primiparity and early reproduction. We then investigate the possible association of early reproduction with lifetime growth and reproductive success later in life. We predicted that females that experienced favorable early-life conditions, were larger, and in better body conditions as subadults would reproduce earlier and more frequently as young adults. We hypothesized that females with greater reproductive allocation as young adults would have slower subsequent growth, reach smaller asymptotic sizes, but have greater reproductive success than those prioritizing early-life growth, after accounting for resource availability and individual heterogeneity in its acquisition and allocation. This research builds upon work by Quesnel et al. (2018) to first explore factors influencing early primiparity and reproduction, then how early-life morphology and environmental conditions affect late life reproductive success.

## Methods

### Study area

We monitored a population of eastern Gray kangaroos in Wilsons Promontory National Park, Victoria, Australia (38°57′S, 146°17′E) from 2008 to 2023. The study area is a 110-ha decommissioned airfield with grasses, ferns, sedges, and shrubs that have encroached on kangaroo habitat over the course of the study (Davis et al. 2010). The climate is temperate, with total annual precipitation of 518 to 1149 mm (Table S1). Winters are cool (June-August average coldest temperature = 0.8 °C, Table S1) and summers are warm (December-February average hottest temperature = 38.7 °C, Table S1). We used the Shallow Inlet (38.63° S, 145.81° E; 20 km north of the study area) and Pound Creek (38.63°S, 145.81° E; 55 km northwest of the study area) weather stations of the Australian Bureau of Meteorology (http://www.bom.gov.au/climate/data/) to obtain climate data.

Seasonal palatable vegetation was estimated since autumn 2009. Vegetation from 50 circular exclosures (0.25m^2^), which excluded herbivores rabbit-sized and larger, was cut, sorted into palatable and unpalatable to kangaroos, oven dried, and weighed (Bergeron et al. 2023). Dry weight of palatable vegetation was divided by exclosure surface area and averaged across seasons, and all exclosures were summed to determined annual available forage in kg/ha. Population density (kangaroo/ha), averaged annually, was estimated through seasonal line-transect sampling (Glass et al. 2015) and DISTANCE software (Thomas et al. 2010). Annual population density estimates ranged from 2.1 to 6.1 kangaroos/ha.

We divided available forage by population density annually to estimate the amount of vegetation per kangaroo. To account for long-term environmental conditions, we calculated the cumulative sum of vegetation per kangaroo, from age 1 to each age tested in select models (Table S2).

### Captures and measurements

Female kangaroos were anesthetized with Zoletil injection via a jabstick, then marked with colored collars and ear tags, and weighed with a spring scale to the nearest 0.25 kg (King et al. 2011). One hind leg was measured with a retractable tape to the nearest mm. Reproductive status was assessed by checking the pouch for young or evidence of lactation. Pouch young weighing 1 kg or more were tagged with small plastic ear tags. Body measurements of pouch young were compared to growth models of captive kangaroos to estimate birthdate (Poole et al. 1982; MacKay et al. 2018). Individuals initially captured as subadults (aged 1 to 2 yr) were assigned to a cohort based on mass and size. Females of unknown age were excluded from analyses. The average annual recapture rate of surviving marked adult females was 88% excluding the 2 yr of the COVID-19 pandemic, when it was about 50%.

The large pouch young (LPY) stage, between 7 to 10 mo of age, is the costliest stage of reproduction as it combines peak lactation and transport of a young that can weigh up to a quarter of maternal mass (Poole 1982; Cripps et al. 2011; Gélin et al. 2016a). Female reproductive success was assessed based on offspring survival to the LPY stage at 7 mo of age. Field observations of marked individuals were also used to determine survival from October 1st to September 30th of the following year (Bergeron et al. 2023). Reproductive success was measured annually, as over 99% of females have one reproductive attempt per year.

### Statistical analysis

We explored predictors and effects of early-life reproduction through 6 models (Table S2). The first three focus on the predictors of early-life reproduction, and the last three focus on the effects of early-life reproduction on later life. We defined early reproduction as reproductive success between ages 3 to 5 as most post-maturation growth occurred at these ages (Figure S1). We fit these models using the “brms” package, version 2.20.4 (Bürkner 2017). We used four independent Markov chains, with 2,500 warmup and 2,500 post-warmup iterations in all models except for the non-linear von Bertalanffy growth model (Model VI) which required 5,000 of each (Table S2). We assessed convergence with scale reduction factors and used bulk and tail effective sample sizes to assess parameter estimate stability (Brooks and Gelman 1998). All continuous variables were scaled to account for differences in magnitudes (Marquardt 1980; Schielzeth 2010). Analyses were conducted in R version 4.2.3via RStudio 2023.12.1-402 (R Core Team 2023; Posit Team 2024).

The annual female recapture rate was over 88% (excluding COVID years; Forrester et al. 2024); only females caught and measured in each year were included, which partially explains the sample size variation across models (see below). All models included morphological and environmental variables to investigate the predictors and effects of early reproduction.

We followed Peig and Green’s (2010) relative condition ($K_{n}$) to estimate female body condition to avoid collinearity between leg length and mass (Figure S2). Our approach was similar to Montana et al. (2020) but used a Bayesian regression of log mass on log leg length ($L_{i})$, using the entire data set, then extracted the mean predicted intercept (*a*) and slope (*b*) to calculate the predicted mass. $K_{n}$ was calculated as the ratio of observed mass ($M_{i}$) to predicted mass ($M_{p}=a\;{L_{i}}^{b}$). We calculated the annual juvenile survival of the study population as the proportion of young of marked mothers that survived from the LPY stage to weaning. Annual juvenile survival served as a proxy for variation in annual environmental conditions and reproductive success, similar to Chambert et al.’s (2014) use of to the number of seal pups born as a surrogate for environmental conditions. Relative condition and size of females aged 2 to 10 were independent of annual juvenile survival (Figures S3-S4). We included cohort as a random effect in all models to capture unmeasured variation among cohorts.

### Predictors of early reproduction

To explore how early environmental conditions and female morphology affected reproduction probability in the subsequent year at ages 2 to 5, we used two generalized linear mixed models (GLMM) with Bernoulli response variables (Models I-II; Table S2). Reproduction is costly, but age 2 kangaroos are not yet reproductive, necessitating separate models (Clutton-Brock et al. 1983). Model I included only age 2 females, while model II included ages 3 to 5. Model I explored how traits at age 2 influence reproduction at age 3, the earliest age of reproduction. Model II included ages 3 to 5 and accounted for the cost of reproduction using a Bernoulli covariate for current reproductive success (yes/no). Model II included a random effect for ID to account for pseudo-replication and interactions between all covariates and age to assess how predictors changed with age (Models I-II; Table S2). Both models included leg length and relative condition as fixed effects, two variables associated with increased reproductive success in young females, annual vegetation per kangaroo as a measure of resource availability, annual juvenile survival of the entire population as a measure of environmental suitability, and cohort as a random intercept to account for cohort effects (Quesnel et al. 2018). To see how these effects shifted with age, we included an interaction between age and all Model II covariates.

Model III was a linear mixed model (LMM) with a Gaussian response variable to explore how subadult (age 2) environmental and morphological conditions affected total reproduction in females between ages 3 to 5 (Table S2). Leg length and relative condition at age 2 were included as fixed effects. We included mean annual juvenile survival and the cumulative sum of vegetation per kangaroo at ages 1 to 2 to test how environmental suitability and subadult resource availability affected early reproduction, respectively. Age 0 was not included as much of the first year is spent in the pouch, highly dependent on maternal care.

Our priors reflected previous work indicating positive effects of size, condition, resource availability, and environmental conditions on the probability of subsequent reproduction (McLoughlin et al. 2006; Quesnel et al. 2018; Toni et al. 2020; Plaisir et al. 2022). To address possible subjectivity or uncertainty in our priors’ directionality, we used narrow, loose, and uninformative priors with no sign constraint (Table S3). We tested each model’s fit and sensitivity to the priors using posterior predictive sensitivity (Gabry et al. 2019). We used prior distributions centered on negative values for the effect of current reproduction to reflect its cost in Models II-III (Table S3; Gélin et al. 2015).

### Effects of early reproduction

To explore potential trade-offs between early reproduction and late-life reproduction, we used two GLMMs (Models IV-V), and a hierarchical nonlinear growth model to investigate the effects of early reproduction on lifetime growth (Model V; Table S2). Ages 6 to 7 were chosen to maximize sample size. Although selective mortality likely introduced some bias, annual survival is over 94% and 86% to 90% for females aged 3 to 6 and 7 to 9, respectively (Bergeron et al. 2023). Females aged 6 to 7 likely allocate more resources to reproduction over growth (Figures S1 and S5; Quesnel et al. 2018).

Both models (VI-VII) used a Bernoulli response variable for annual reproduction (yes/no) for ages 6 to 7. Model IV included a continuous fixed effect for early reproduction between ages 3 to 5 as a predictor of reproductive allocation, while Model VII incorporated early primiparity at age 3 (yes/no) as a binary predictor for early reproductive allocation (Table S2). We included continuous fixed effects for leg length and $K_{n}$ at age 5 in both models to assess how these indirect measures of fitness affected late-life reproduction. The cumulative vegetation per kangaroo and the average juvenile survival between ages 1 to 5 were included to account for long-term environmental conditions experienced in early life. Early primiparity and increased cumulative reproduction are costly, but may also indicate higher reproductive potential (Descamps et al. 2006; Weladji et al. 2008; Moyes et al. 2011; Fay et al. 2016; Forrester et al. 2024). Because we were unsure of the direction of the effect of early reproduction, we tested the sensitivity of the model using four different prior distributions: uninformative, narrow negative, narrow positive, and a loose priors centered at zero for early reproduction and primiparity (Table S3). We used narrow positive priors for size, relative condition, and the cumulative vegetation per kangaroo, consistent with previous models (Table S3).

To quantify the effect of early reproduction on lifetime growth, we used a Bayesian hierarchical non-linear model of a modified von Bertalanffy growth function (vBGF; Model VI; Tables S2-S3), following Vincenzi et al. (2016) and Shelton and Mangel (2012), where the expected leg length in mm (*L*) of individual (*i*) in cohort (*j*) at age in years (*t*) is


$$\begin{array}{l} L_{ij}\left(t\right)=L_{0ij}e^{-k_{i}t}+\;\gamma_{ij}\;{k_{ij}}^{\left(\psi -1\right)}\left(1-e^{-k_{i}t}\right). \end{array}$$ (1)

Model VI integrates both fixed and random effects to explore how initial leg length ($L_{0ij}$), environmental factors ($\gamma_{ij}$), and individual differences ($k_{ij}$) affected individual size ($L_{ij}$; Pilling et al. 2002; Helser and Lai 2004; Cafarelli et al. 2019). The metabolic scaling parameter $\left(k_{ij}\right)$ captures phenotypic capacities for growth, either behavioral or physiological, while the environmental-behavioral interaction parameter (ψ) modulates the degree to which an individual’s realized anabolism depends on environmental versus individual factors, at the population level (Vincenzi et al. 2016). When ψ = 0, individual growth is solely determined by environmental factors (γ). When ψ = 1, growth is solely determined by individual physiological and behavioral traits (*k*) that influence resource acquisition and allocation (Vincenzi et al. 2016). We included initial leg length, the first parameter in the growth curve, based on pouch young measurements, as a starting point.

To ensure leg growth was positive, we used a log-link function on the vBGF parameters: initial leg length ($L_{0ij}$), the metabolic scaling parameter ($k_{ij}$) and environmental parameter ($\gamma_{ij}$).


$$\begin{array}{l} L_{ij}\left(t\right)=e^{ln(L_{0ij})}\cdot e^{-ln\left(k_{ij}\right)t}+e^{\ln\left(\gamma_{ij}\right)\;\cdot \;ln{\left(k_{ij}\right)}^{\left(\psi -1\right)}}\cdot (1-e^{e^{-ln\left(k_{ij}\right)}t}) \end{array}$$

$$\begin{array}{l} \begin{array}{llll} & L_{0ij}=\beta_{0L_{0}}+\;\beta_{1L_{0}}X_{1}+b_{L_{0i}}+b_{L_{0j}}\; & \;\;k_{ij}=\;\beta_{0k}+\;\beta_{1k}X_{1\;}+\;b_{k_{i}}+b_{k_{j}}\;\;\;\; & \;\gamma_{ij}=\;\beta_{0\gamma }+\;\beta_{1\gamma }X_{2\;}+\;b_{\gamma_{i}}+b_{\gamma_{j}}\; \end{array} \end{array}$$ (2)

As most growth after sexual maturity occurs from ages 3 to 6 (Figure S1), we tested how early reproduction, as a continuous fixed effect ($X_{1})$, varied with $k_{ij}$ and $L_{0ij}$. We included vegetation per kangaroo ($X_{2})$ to determine how annual resource abundance and population density affected the $\gamma_{ij}$. We included random intercepts for ID ($b_{i})\;$and cohort ($b_{j})$ in the estimation of all three parameters to account for unmeasured variation explained by individual heterogeneity and cohort effects (Lindström 1999; Hamel et al. 2018).

We explored the relationship between early reproduction effort and initial leg length at age 0, measured when females were at the LPY stage. Larger initial sizes should indicate greater maternal care or silver spoon effects. We chose priors for the environmental, metabolic scaling, and the initial leg length parameter by visualizing prior predictive distributions and choosing values that covered a wide range of biologically-plausible growth curves that asymptote around age 10, where females reach survival senescence (Table S3; Gabry et al. 2019; Bergeron et al. 2023). We used a beta distribution for the environmental-behavioral interaction parameter (Table S3; ψ). We chose a mean for this beta distribution of 0.5 and a standard deviation of 0.5 to express our uncertainty in the relative importance of environmental and or individual factors on growth (Table S3).

## Results

### Predictors of early reproduction

**Fig. 1.**
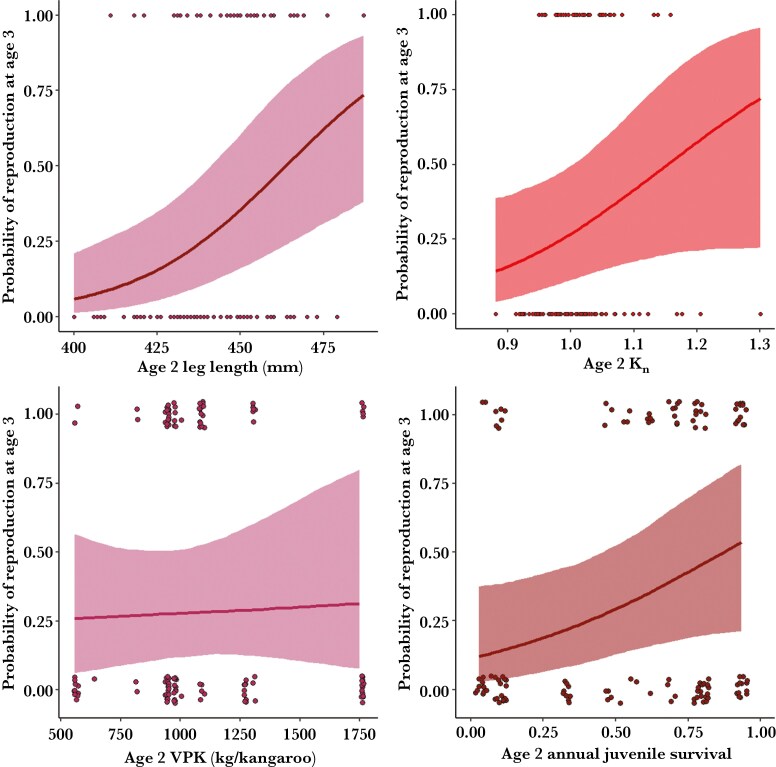
Mean predicted conditional effect (colored lines) and 95% credible intervals (shades) of morphological and environmental covariates measured at age 2 (dots) on the probability of reproduction at age 3 for 102 female eastern Grey kangaroos in 2010 to 2022, Wilsons Promontory National Park. Covariates include leg length at a proxy for size (top left), relative female condition (Kn; top right), vegetation per kangaroo (VPK; bottom left) and annual population-level juvenile survival as a proxy for environmental suitability (bottom right).

**Fig. 2.**
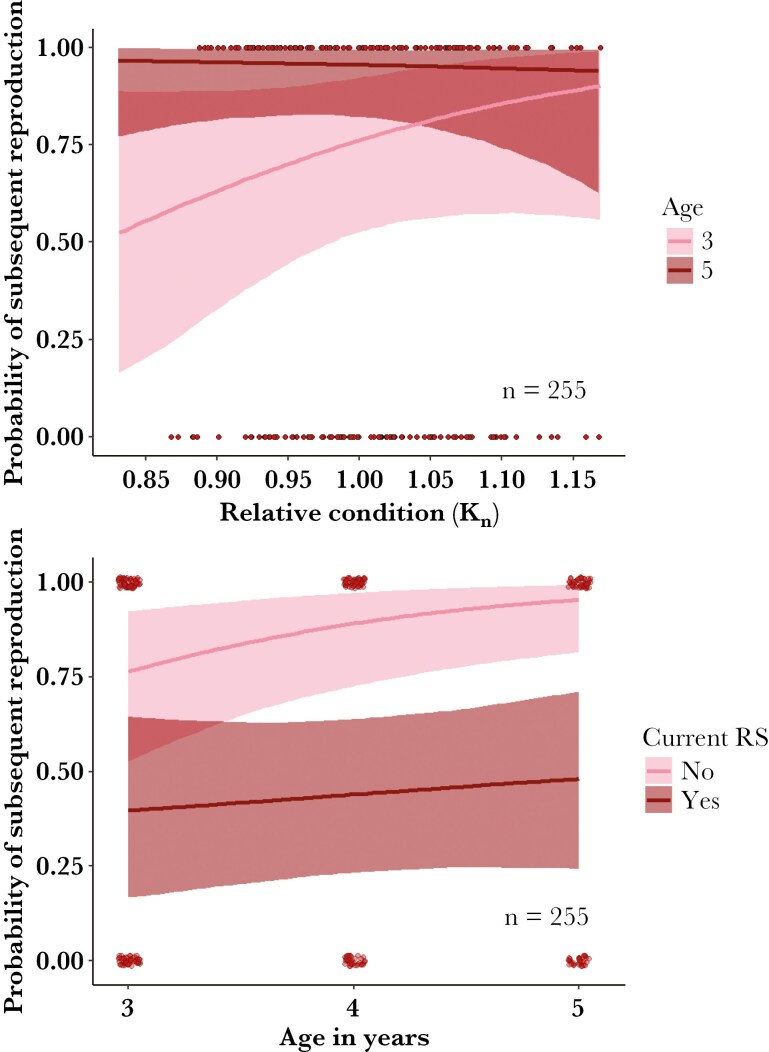
Mean predicted conditional effect (colored lines) and 95% credible intervals (shades) of relative condition (Kn; top) and current reproductive success (RS; bottom) on the probability of reproduction in the following year for female eastern Grey kangaroos aged 3 to 5 yr in 2010 to 2022, Wilsons Promontory National Park.

Both early-life size and body condition positively influenced early reproduction and primiparity. We used positive narrow priors for Model I-III of the predictors of early-life reproduction as their predicted estimates were not sensitive to priors (Figures S6-S8, Table S4). Models I-II, which explored predictors of subsequent reproduction at ages 2 to 5, indicated that the strength of the positive effect of early-life morphology and environment declined with age (Figs 1-2, Tables S4-S5). Leg length had the strongest predicted effect on subsequent-year reproduction at age 2, and there was no evidence to suggest an effect of size on reproductive success, nor an interaction with age for ages 3 to 5 (Tables S4-S5). A 2-yr-old female with a leg length of 425 mm (2 standard deviations below the mean; Fig. 1) had a predicted mean probability of primiparity at age 3 of 11% (95% CrI: [0.02, 0.28]). That probability increased to 33% (95% CrI: [0.14, 0.57]) at 442 mm (mean leg length), and 68% (95% CrI: [0.38, 0.90) at 459 mm (2 standard deviations above the mean; Fig. 1). Similarly, the positive effect of relative condition ($K_{n}$) was most pronounced at age 2 and decreased between ages 3 to 5, as suggested by its interaction with age (Figs 1-2, Tables S4-S5). A 2-yr-old female with a $K_{n}$ of 0.9 (2 standard deviations below the mean $K_{n}$) had a predicted probability of primiparity at age 3 of 18% (95% CrI: [0.05, 0.44]) and one with $K_{n}$of 1.08 (2 standard deviations above the mean) had a mean probability of 53% (95% CrI: [0.22, 0.81]; Figure S12). At age 3 to 5, females with a $K_{n}$of 0.93 (2 standard deviations below the mean $K_{n}$) had a mean probability of subsequent reproduction of 57% (95% CrI: [0.24, 0.88]) and 95% (95% CrI: [0.81, 0.99]), respectively, while females with a $K_{n}$of 1.09 (2 standard deviations above the mean $K_{n}$) had a mean probability of subsequent reproduction of 86% (95% CrI: [0.57, 0.98]) and 82% (95% CrI: [0.67, 0.99]; Fig. 2), respectively. Current reproductive effort reduced subsequent probability of reproductive success, with its effect also weakening with age (Fig. 2, Tables S4-S5). Population-level juvenile survival had a positive effect on subsequent reproduction in 2-yr-old females, but there was weak evidence to suggest this effect varied with age between ages 3 to 5 (Fig. 1, Table S4-S5). There was little effect of vegetation per kangaroo on subsequent reproduction at age 2, and weak evidence of a positive effect of vegetation per kangaroo that varied with age between ages 3 to 5 (Tables S4-S5). There was notable cohort-level variation at age 2, while individual identity explained much of the variation in subsequent reproduction between ages 3 to 5 (Figure S9-S10, Tables S4-S5).

**Fig. 3.**
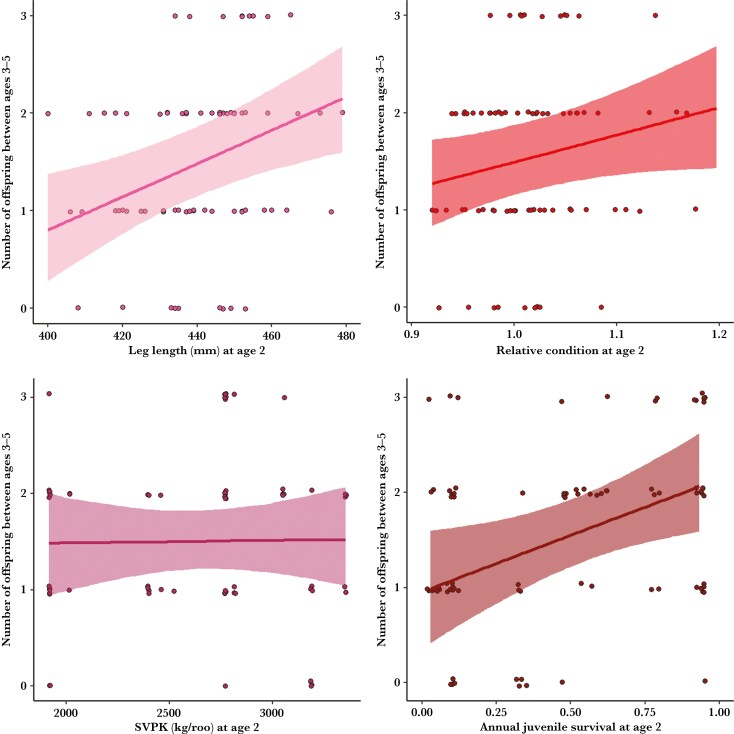
Mean conditional predicted effects of leg length, relative condition, subadult vegetation per kangaroo (SVPK) and juvenile survival in the year the female was age 2, with 95% credible intervals, on the number of offspring between ages 3 to 5, with points indicating raw data, for 72 females between 2010 to 2022 in Wilsons Promontory National Park.

Model III, which explored the predictors of early-life reproduction, suggested that age 2 females that were larger, in better condition, and experienced years of high juvenile survival had greater reproductive success at ages 3 to 5 (Fig. 3, Table S5). Subadult cumulative vegetation per kangaroo did not influence early-life reproduction (Fig. 3). There was little variation between cohorts (Table S5). At age 2, a small female with a leg length of 412 mm or that experienced a year of poor juvenile survival (4%) was predicted to have 1 offspring between ages 3 to 5, while a large 2-yr-old female with a leg length of 470 mm or that experienced a year of high juvenile survival (87%) was predicted to have 2 offspring between ages 3 to 5 (Table S5).

### Effects of early reproduction

**Fig. 4.**
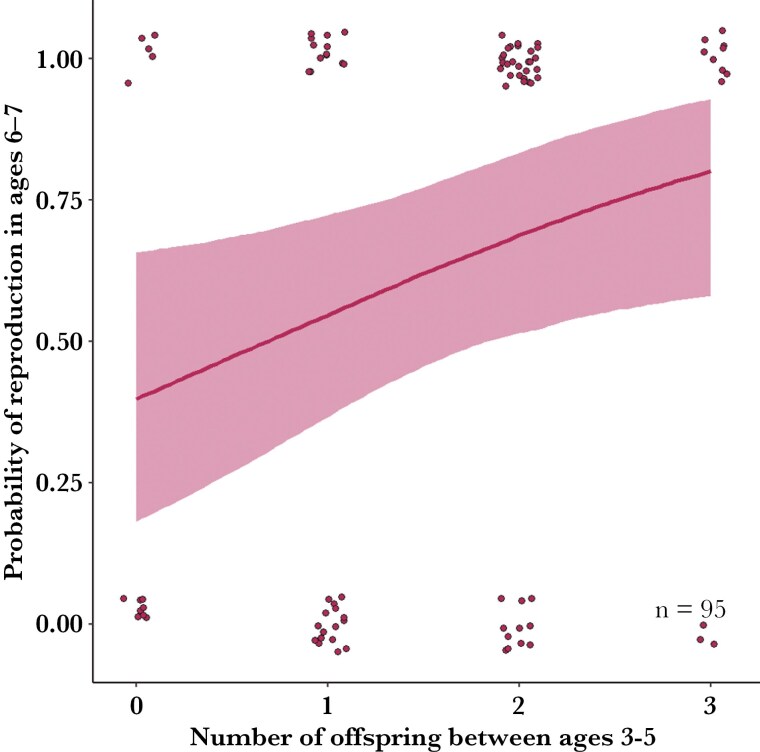
Mean conditional predicted effect (red line) and 95% credible interval (red shade) of reproductive success between ages 3 to 5 on the probability of reproduction at ages 6 to 7 for female eastern Grey kangaroos, 2010 to 2018 in Wilsons Promontory National Park. Red points represent raw data.

Narrow negative priors for early-life reproduction and early primiparity resulted in a positive effect on reproduction at ages 6 to 7. Consequently, we selected models with narrowly constrained positive priors based on limited sensitivity to the priors and differences in predictive power (Figures S11-S12). We found a positive effect of early-life reproductive success on reproduction at ages 6 to 7; females that did not reproduce between ages 3 to 5 had a predicted probability of subsequent reproduction of 34% (95% CrI: [0.13, 0.61]) and 49% (95% CrI: [0.23, 0.76]) at ages 6 to 7, respectively, compared to 73% (95% CrI: [0.49, 0.91]) and 84% (95% CrI: [0.64, 0.95]) for females that reproduced every year between ages 3 to 5 (Fig. 4, Table S5). There was weak evidence to suggest a positive effect of cumulative vegetation per kangaroo, mean juvenile survival, and leg length at age 5 on reproductive success between ages 6 to 7 (Table S5), and no evidence for an effect of Kn. Early primiparity was also associated with greater reproduction at 6 to 7 yr of age (Figure S13).

**Fig. 5.**
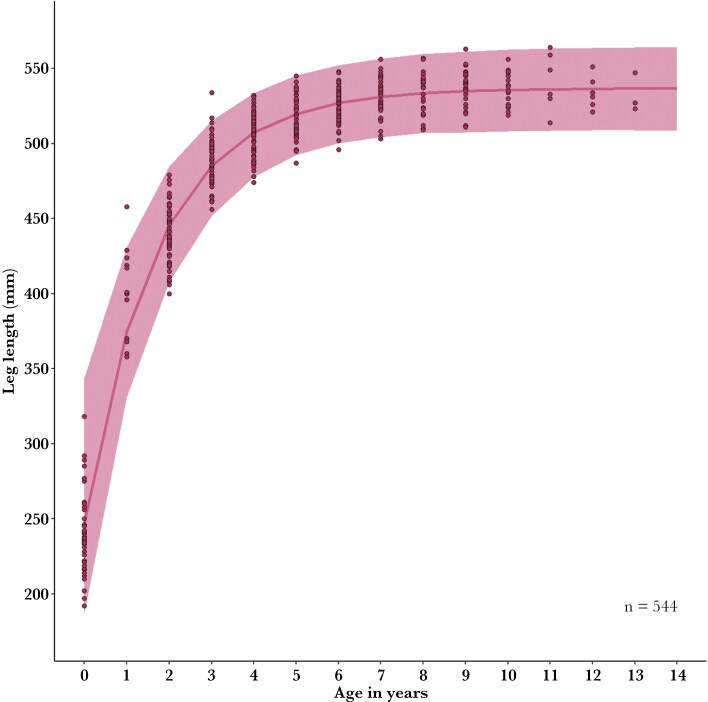
Mean predicted growth curve of female kangaroos. The raw data (dots) include 544 leg measurements for 92 females in 2010 to 2022, Wilsons Promontory National Park, Victoria, Australia.

**Fig. 6.**
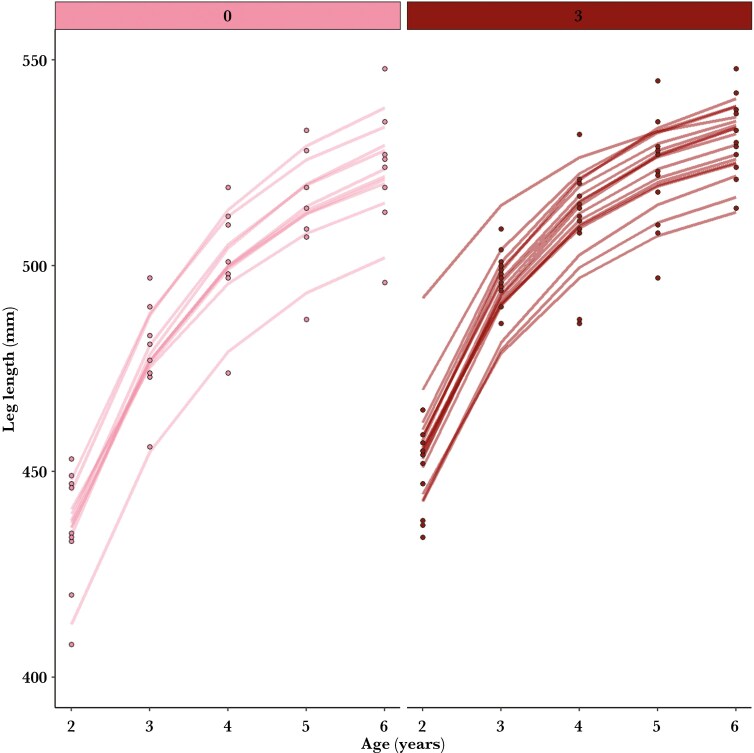
Mean predicted growth curves of 27 female eastern Grey kangaroos that produced 0 or 3 offspring that survived to 7 mo of age between ages 3 and 5 with raw leg lengths (dots) measured in 2010 to 2022, Wilsons Promontory National Park.

At the population level, the von Bertalanffy growth function exhibited a good fit to the raw data (Fig. 5). Associations between early reproductive efforts and subsequent reproductive success are subtle when viewed across ages 0 to 14 (Figs 6, S13-S14), but are more apparent at younger ages (see below). Most raw leg measurements fell within 95% of the predicted credible intervals. Vegetation per kangaroo had no effect on the environmental parameter (Table S5). Opposite to our predictions, early-life reproduction had a small positive effect on initial growth. Females with no reproduction between ages 3 to 5 were smaller as subadults but grew faster to catch up to larger counterparts by age 6. There was substantial uncertainty in this prediction (Figure S14-S15, Table S5).

Individual identity and cohort explained more variation in growth and initial size than early-life reproduction (Table S5). By visualizing the predicted growth curves of individual females rather than mean predicted growth curves, we found differences in early-life growth among females according to early-life reproductive efforts (Figure S16). For example, comparing females that had 0 or 3 offspring surviving to 7 mo when aged 3 to 5 yr, Fig. 6 shows how the latter were larger at ages 2 to 3 but the size difference mostly disappeared by age 6. Early-life reproduction generally increased with greater values of the metabolic scaling parameter, initial leg length, and the environmental parameter in the von Bertalanffy growth curve (Figures S17-S19). The model predicted a mean initial leg length of 245 mm for LPYs and a mean ψ value of 0.93 (Table S5).

## Discussion

Our 13-yr study of eastern Gray kangaroos revealed morphological and environmental predictors of primiparity and early-life reproduction. Females that were larger and in better condition as 2-yr-olds experienced immediate fitness gains through earlier primiparity and greater reproduction when aged 3 to 5 yr. This result aligns with previous research indicating higher fecundity in tall young female kangaroos and earlier reproduction in larger, heavier mammals (Green and Rothstein 1991; Parker et al. 2017; Quesnel et al. 2018; Kalberer et al. 2019). We suggest the diminishing strength of the positive effect of size and condition on subsequent reproduction with age is likely due to costs of reproduction (Gélin et al. 2015; Toni et al. 2020). As larger 2-yr-olds reproduced at age 3, the costs of that early reproduction likely limited their ability to reproduce again at age 4. That may explain why condition appeared to have the strongest positive effect on subsequent reproduction at age 3: females that were large but had lost condition because of their early reproductive effort at 3 were unable to reproduce at age 4. Females foregoing a reproductive opportunity at ages 3 to 5, and its associated costs, may have higher chances of subsequent reproduction, regardless of size and condition, creating variation in sizes of females reproducing at different ages.

The effect of size and condition on subsequent reproduction decreased in ages 4 to 5, likely due to increased variation in size, condition, and reproductive histories within our sample. Cumulative reproduction decreases subsequent reproduction in female kangaroos (Forrester et al. 2024). Indeed, a small nulliparous 5-yr-old in moderate condition may be more likely to reproduce than a large 5-yr-old in good condition with high cumulative reproduction. Age 2 subadults with the greatest size and condition have a clear fitness advantage through early primiparity, likely due to weaker reproductive costs. This advantage persists through ages 3 to 5, as larger females in good condition are predicted to reproduce once more between ages 3 to 5 than their smaller age 2 counterparts.

Females that experience favorable early-life environmental conditions exhibited greater reproductive success, suggesting silver-spoon effects. Young females had greater early-life reproduction and earlier primiparity if in the year they were 2-yr-olds, juvenile survival was high (Fig. 2). High annual juvenile survival likely reflected favorable environmental conditions that were not detected by our measure of vegetation availability (Plaisir et al. 2022). Previous research in the study population suggested a limited effect of available forage on age-specific survival, although an analysis of survival from 10 to 21 mo did find a positive effect of available forage (Plaisir et. al. 2022; Bergeron et al. 2023). Positive effects of favorable early-life environmental conditions are frequently observed in large herbivores (Gaillard et al. 2000; Martin and Festa-Bianchet 2012; Douhard et al. 2013). The greater variation among cohorts in the subsequent reproduction models likely captured annual variation, attributable to the annual timescale of most variables, except for cumulative vegetation per kangaroo, which had negligible effects. The minimal cohort effects in older ages confirm a pattern commonly observed in large herbivores (Hamel et al. 2016). Pre-reproductive size and condition confer an early fitness advantage, while a combination of favorable environmental conditions and greater individual resource acquisition likely offsets early reproductive costs.

We found limited effects of early-life reproduction on growth and reproduction in late life. Contrary to our predictions, females with greater early-life reproduction and earlier primiparity exhibited greater reproduction between ages 6 to 7. Higher early-life reproduction was likely associated with greater reproductive potential that persisted at later ages. Earlier age at first reproduction is associated with greater reproductive performance and adult survival in long-lived Wandering Albatrosses (*Diomedea exulans*), and greater total calf production in moose (*Alces alces*), suggesting that age of primiparity is a good predictor of individual reproductive potential (Fay et al. 2016; Markussen et al. 2018).

Animal growth is typically sigmoidal in shape, often modeled using non-linear growth equations (Zullinger et al. 1984; Gilg 2000). While polynomial functions can provide a better fit, they often lack a clear biological meaning and have limited applications compared to non-linear growth models (Chen et al. 1992; Lugert et al. 2016). The vBGF, based on anabolism and catabolism, facilitates testing of biologically relevant questions in energy allocation (von Bertalanffy 1957). It has been used to model growth of large mammals like polar bears (*Ursus maritimus*), Asian elephants (*Elephas maximus*), and white-tailed deer (*Odocoileus virginianus*; Derocher and Wiig 2002; Monteith et al. 2009; Mumby et al. 2015). There is considerable debate over random effect structure and parameterization within the vBGF. Vincenzi et al. (2016) argue that the physiological processes that affect k and $L_{\infty }$ parameters are similar and treating them independently lacks physiological and biological interpretation. They suggest Bayesian estimation, with repeat measures, offers flexibility and insights into shared and individual effects on growth in natural populations (Vincenzi et al. 2014). Bayesian estimation yielded a biological meaningful and well-fitting female eastern Gray kangaroo growth curve.

Growth and reproduction varied substantially among cohorts and individuals. In individuals, this could be driven by unmeasured effects including physiological differences, human disturbance, environmental tolerance, parasite resistance,) and acquisition, progression and transmission of disease (Gabriel et al. 2005; Biro and Stamps 2010; Strasser and Heath 2013; Hayward et al. 2014; McDonald et al. 2018). The positive mean effect of early-life reproduction on reproduction in ages 6 to 7 reinforces the notion that females with greater reproductive potential early in life tend to maintain that reproductive advantage in later years, despite negligible size differences. This suggestion is supported by the environmental-behavioral interaction parameter of 0.94, suggesting that individual growth is strongly dependent on individual physiological and behavioral traits (*k*), influencing resource acquisition and allocation (Vincenzi et al. 2016). There are size and growth differences between females with high and low early-life reproduction; the latter start smaller, forgo early reproduction, grow faster, and catch up by age 6, at the cost of reduced reproduction over several years. Yearsley et al. (2004) suggested that delayed costs of growth can be associated with adaptive responses to growth limitation, shifting growth costs onto future reproduction. Compensatory growth has been found to reduce reproductive success in guppies (*Poecilia reticulata*; Auer et al. 2010). The skeletal growth of kangaroo females appears to asymptote at 7 to 9 yr (Fig. 4), but their substantial growth after maturity enables them to navigate trade-offs between reproduction, for immediate fitness gains, or growth, for future fitness potential, across much of their life. In species with indeterminate growth, young females generally prioritize growth (Folkvord et al. 2014). A limitation of our study is the lack of data on reproductive or survival senescence, as many females we monitored are still alive. Increased allocation to early-life reproduction could result in senescence of maternal performance (Nussey et al. 2006; McKenna-Ell et al. 2023). Given the complex interplay of growth and reproduction, further investigation using path analysis could provide more insight into the causal structure of this system, effect sizes and directionality, as well as unmeasured variables.

Our research revealed a strong importance of subadult size and silver-spoon effects on fitness, which resulted in increased reproduction over several years. Females with high early-life reproduction benefit from large subadult size and likely greater resource acquisition and allocation potential, prioritizing reproduction over growth. Similarly, earlier reproduction in bison (*Bison bison*) reduced growth in the subsequent year but led to higher fecundity in the long term (Green and Rothstein 1991). However, by age 6, size differences wane, suggesting flexible navigation of growth-reproduction trade-offs in a large, long-lived iteroparous mammal wwith indeterminate growth. Individual heterogeneity in long-lived species can be shaped by early-life size and condition, environmental variables, and unmeasured covariates, all of which can influence key fitness parameters and can have substantial impacts on population dynamics (Plard et al. 2015; Forrester et al. 2024).The relationship between size at age 2 and reproduction in ages 3 to 5 reveals a pattern where smaller individuals focus on growth, while larger females prioritize reproduction, similar to van Noordwijk and De Jong‘s (1986) rich family, poor family analogy; the rich get richer, and the poor stay poor.

## Supplementary Material

araf017_suppl_Supplementary_Material

## Data Availability

Analyses reported in this article can be reproduced using the data provided by Forrester et al. (2025).
