# Supplementary material for: Silver spoons, reproduction, and growth catch-up in eastern Grey kangaroos
Source: Behav Ecol. 2025 Feb 26;36(3):araf017. doi: 10.1093/beheco/araf017 (PMC11933691; doi:10.1093/beheco/araf017)
Supplement: araf017_suppl_Supplementary_Material [file araf017_suppl_supplementary_material.docx]

**Supplementary material for “Silver spoons, reproduction, and growth catch-up in eastern grey kangaroos”**

# **Tables**

Supplementary Table 1. Annual sum of precipitation (mm) and means of daily minimum and maximum temperature (°C) in winter (June to August) and summer (December to February). Precipitation and temperature data taken from Shallow Inlet and Pound Creek, respectively, Australian Bureau of Meteorology (http://www.bom.gov.au/climate/data/)

| Year | Total rainfall | Winter mean maximum temperature | Summer mean maximum temperature | Winter mean minimum temperature | Summer mean minimum temperature |
| --- | --- | --- | --- | --- | --- |
| 2008 | 703.50 | 13.38 | 24.55 | 5.14 | 12.17 |
| 2009 | 870.20 | 14.68 | 23.97 | 7.04 | 11.01 |
| 2010 | 1031.40 | 13.65 | 24.70 | 5.98 | 12.22 |
| 2011 | 1148.80 | 14.65 | 23.26 | 6.33 | 12.80 |
| 2012 | 968.80 | 14.06 | 24.08 | 6.28 | 12.48 |
| 2013 | 1017.60 | 14.88 | 24.57 | 7.29 | 12.41 |
| 2014 | 618.40 | 14.55 | 24.82 | 6.29 | 12.61 |
| 2015 | 517.80 | 13.31 | 24.01 | 5.55 | 12.84 |
| 2016 | 819.00 | 14.62 | 25.69 | 6.68 | 12.67 |
| 2017 | 917.60 | 14.18 | 24.27 | 5.52 | 12.32 |
| 2018 | 744.00 | 14.18 | 25.19 | 6.10 | 13.25 |
| 2019 | 804.40 | 14.04 | 25.25 | 6.80 | 13.58 |
| 2020 | 911.40 | 14.26 | 23.36 | 5.69 | 12.41 |
| 2021 | 876.90 | 14.79 | 22.90 | 6.81 | 11.68 |
| 2022 | 871.50 | 14.49 | 24.64 | 5.98 | 12.95 |
| 2023 | 703.20 | 15.04 | 24.22 | 7.49 | 12.59 |

Supplementary Table 2. Model structures for the predictors and effects of early reproduction with fixed effects including relative condition ($K_{n}$), vegetation per kangaroo (VPK), cumulative vegetation per kangaroo (CVPK), and subadult vegetation per kangaroo (CVPK). Models for later life reproductive success include early-life reproduction (ER) and early primiparity (EP). Model VI based on (2) in the main text, with parameters (*) based priors defined in Table S3.

| Section | Model | Response variable | Fixed effects | Random intercepts |
| --- | --- | --- | --- | --- |
| Predictors of early reproduction | I – Age 2 | Probability of subsequent reproduction | Leg length + $K_{n}$+ VPK + Annual juvenile survival | Cohort |
| Predictors of early reproduction | II – Age 3-5 | Probability of subsequent reproduction | Leg length*Age + $K_{n}$*Age + VPK*Age + Annual juvenile survival*Age + Current reproduction*Age | Cohort + ID |
| Predictors of early reproduction | III | Early reproduction total | Leg length at age 2 + $K_{n}$ at age 2 + CVPK at age 2 + Mean juvenile suvival at age 2 | Cohort |
| Effects of early reproduction | IV | Probability of reproduction in ages 6-7 | Age + Early-life reproduction + Leg length at age 5 + $K_{n}$ at age 5 + CVPK to age 5 + Mean juvenile suvival to age 5 | Cohort + ID |
| Effects of early reproduction | V - EP | Probability of reproduction in ages 6-7 | Age + Early primiparity + Leg length at age 5 + $K_{n}$ at age 5 + CVPK to age 5 + Mean juvenile suvival to age 5 | Cohort + ID |
| Effects of early reproduction | VI | Leg length | Initial size* + Metabolic scaling parameter* + Environmental parameter* | ID + Cohort |

Supplementary Table 3. Prior specification for all models’ parameters, with their class, distribution, means, standard deviations, and rate parameter. All continuous variables are scaled, and all parameters for model VI are log-transformed.

| **Model I** | | | | | | | | | | | | |
| --- | --- | --- | --- | --- | --- | --- | --- | --- | --- | --- | --- | --- |
| Prior | | Parameter | | Class | Distribution | | Mean (μ) | | Standard deviation (σ) | | | |
| Narrow positive | | Leg length | | Slope | Normal | | 0.5 | | 0.5 | | | |
|  | | Juvenile survival | | Slope | Normal | | 0.5 | | 0.5 | | | |
|  | | Relative condition | | Slope | Normal | | 0.5 | | 0.5 | | | |
|  | | Vegetation per kangaroo | | Slope | Normal | | 0.5 | | 0.5 | | | |
|  | | Cohort | | Standard deviation | Normal | | 0 | | 2.5 | | | |
| Loose positive | | Leg length | | Slope | Normal | | 1 | | 1 | | | |
|  | | Juvenile survival | | Slope | Normal | | 1 | | 1 | | | |
|  | | Relative condition | | Slope | Normal | | 1 | | 1 | | | |
|  | | Vegetation per kangaroo | | Slope | Normal | | 1 | | 1 | | | |
|  | | Cohort | | Standard deviation | Normal | | 0 | | 2.5 | | | |
| Uninformative (flat) | | Leg length | | Slope | Uniform | | - | | - | | | |
|  | | Juvenile survival | | Slope | Uniform | | - | | - | | | |
|  | | Relative condition | | Slope | Uniform | | - | | - | | | |
|  | | Vegetation per kangaroo | | Slope | Uniform | | - | | - | | | |
|  | | Cohort | | Standard deviation | Student t-test | | 0 | | 2.5 | | | |
| **Model II** | | | | | | | | | | | | |
| Prior | Parameter | | Class | | | Distribution | | Mean (μ) | | Standard deviation (σ) | | |
| Narrow positive | Leg length*Age | | Slope | | | Normal | | 0.5 | | 0.5 | | |
|  | Juvenile survival*Age | | Slope | | | Normal | | 0.5 | | 0.5 | | |
|  | Relative condition*Age | | Slope | | | Normal | | 0.5 | | 0.5 | | |
|  | Vegetation per kangaroo*Age | | Slope | | | Normal | | 0.5 | | 0.5 | | |
|  | Current reproductive success*Age | | Slope | | | Normal | | -1 | | 0.5 | | |
|  | Cohort | | Standard deviation | | | Normal | | 0 | | 2.5 | | |
| Loose positive | Leg length*Age | | Slope | | | Normal | | 1 | | 1 | | |
|  | Juvenile survival*Age | | Slope | | | Normal | | 1 | | 1 | | |
|  | Relative condition*Age | | Slope | | | Normal | | 1 | | 1 | | |
|  | Vegetation per kangaroo*Age | | Slope | | | Normal | | 1 | | 1 | | |
|  | Current reproductive success*Age | | Slope | | | Normal | | -1 | | 1 | | |
|  | Cohort | | Standard deviation | | | Student t-test | | 0 | | 2.5 | | |
| Uninformative (flat) | Leg length*Age | | Slope | | | Uniform | | - | | - | | |
|  | Juvenile survival*Age | | Slope | | | Uniform | | - | | - | | |
|  | Relative condition*Age | | Slope | | | Uniform | | - | | - | | |
|  | Vegetation per kangaroo*Age | | Slope | | | Uniform | | - | | - | | |
|  | Current reproductive success*Age | | Slope | | | Uniform | | - | | - | | |
|  | Cohort | | Standard deviation | | | Student t-test | | 0 | | 2.5 | | |
| **Model III** | | | | | | | | | | | | |
| Prior | | Parameter | | Class | Distribution | | Mean (μ) | | Standard deviation (σ) | | | |
| Narrow positive | | Age 2 leg length | | Slope | Normal | | 0.5 | | 0.5 | | | |
|  | | Age 2 juvenile survival | | Slope | Normal | | 0.5 | | 0.5 | | | |
|  | | Age 2 relative condition | | Slope | Normal | | 0.5 | | 0.5 | | | |
|  | | Cumulative vegetation per kangaroo | | Slope | Normal | | 0.5 | | 0.5 | | | |
|  | | Cohort | | Standard deviation | Normal | | 0 | | 2.5 | | | |
| Loose positive | | Age 2 leg length | | Slope | Normal | | 1 | | 1 | | | |
|  | | Age 2 juvenile survival | | Slope | Normal | | 1 | | 1 | | | |
|  | | Age 2 relative condition | | Slope | Normal | | 1 | | 1 | | | |
|  | | Cumulative vegetation per kangaroo | | Slope | Normal | | 1 | | 1 | | | |
|  | | Cohort | | Standard deviation | Normal | | 0 | | 2.5 | | | |
| Uninformative (flat) | | Age 2 leg length | | Slope | Uniform | | - | | - | | | |
|  | | Age 2 juvenile survival | | Slope | Uniform | | - | | - | | | |
|  | | Age 2 relative condition | | Slope | Uniform | | - | | - | | | |
|  | | Cumulative vegetation per kangaroo | | Slope | Uniform | | - | | - | | | |
|  | | Cohort | | Standard deviation | Student t-test | | 0 | | 2.5 | | | |
| **Models IV-V** | | | | | | | | | | | | |
| Prior | | Parameter | | Class | Distribution | | Mean (μ) | | Standard deviation (σ) | | | |
| Narrow positive | | Early reproduction / Early primiparity | | Slope | Normal | | 0.5 | | 0.5 | | | |
|  | | Age | | Slope | Normal | | 0.5 | | 0.5 | | | |
|  | | Age 5 leg length | | Slope | Normal | | 0.5 | | 0.5 | | | |
|  | | Age 5 relative condition | | Slope | Normal | | 0.5 | | 0.5 | | | |
|  | | Cumulative vegetation per kangaroo between ages 1-5 | | Slope | Normal | | 0.5 | | 0.5 | | | |
|  | | Mean juvenile survival between ages 1-5 | | Slope | Normal | | 0.5 | | 0.5 | | | |
|  | | Cohort | | Standard deviation | Normal | | 0 | | 2.5 | | | |
| Narrow negative | | Early reproduction / Early primiparity | | Slope | Normal | | -0.5 | | 0.5 | | | |
|  | | Age | | Slope | Normal | | 0.5 | | 0.5 | | | |
|  | | Age 5 leg length | | Slope | Normal | | 0.5 | | 0.5 | | | |
|  | | Age 5 relative condition | | Slope | Normal | | 0.5 | | 0.5 | | | |
|  | | Cumulative vegetation per kangaroo between ages 1-5 | | Slope | Normal | | 0.5 | | 0.5 | | | |
|  | | Mean juvenile survival between ages 1-5 | | Slope | Normal | | 0.5 | | 0.5 | | | |
|  | | Cohort | | Standard deviation | Normal | | 0 | | 2.5 | | | |
| Loose cenetered | | Early reproduction / Early primiparity | | Slope | Normal | | 0 | | 2 | | | |
|  | |  | |  |  | |  | |  | | | |
|  | | Age 5 leg length | | Slope | Normal | | 0.5 | | 0.5 | | | |
|  | | Age 5 relative condition | | Slope | Normal | | 0.5 | | 0.5 | | | |
|  | | Cumulative vegetation per kangaroo between ages 1-5 | | Slope | Normal | | 0.5 | | 0.5 | | | |
|  | | Mean juvenile survival between ages 1-5 | | Slope | Normal | | 0.5 | | 0.5 | | | |
|  | | Cohort | | Standard deviation | Normal | | 0 | | 2.5 | | | |
| Uninformative (flat) | | Early reproduction | | Slope | Uniform | | - | | - | | | |
|  | | Age | | Slope | Uniform | | - | | - | | | |
|  | | Age 5 leg length | | Slope | Uniform | | - | | - | | | |
|  | | Age 5 relative condition | | Slope | Uniform | | - | | - | | | |
|  | | Cumulative vegetation per kangaroo between ages 1-5 | | Slope | Uniform | | - | | - | | | |
|  | | Mean juvenile survival between ages 1-5 | | Slope | Uniform | | - | | - | | | |
|  | | Cohort | | Standard deviation | Student t-test | | 0 | | 2.5 | | | |
| **Model VI** | | | | | | | | | | | | |
| Prior | | Parameter | | Class | Distribution | | Mean (μ) | | Standard deviation (σ) | | Rate parameter (λ) | |
| Growth parameters | | Initial size | | Slope | Normal | | 5.52 | | 4.61 | | - | |
|  | | Initial size | | Standard deviation | Exponential | | - | | - | | 1 | |
|  | | Metabolic scaling parameter | | Slope | Normal | | 0.3 | | 0.1 | | - | |
|  | | Metabolic scaling parameter | | Standard deviation | Exponential | | - | | - | | 1 | |
|  | | Environmental parameter | | Slope | Normal | | 6.4 | | 0.5 | | - | |
|  | | Environmental parameter | | Standard deviation | Exponential | | - | | - | | 1 | |
|  | | Environmental-behavioural interaction parameter | | Slope | Beta | | 4 | | 4 | | | - |
|  | | Residual standard deviation | | Sigma | Exponential | | - | | - | | | 1 |

Supplementary Table 4. Posterior summary of the predictors of early reproduction models with the Gelman-Rubin statistic (Rhat), bulk and tail effective sample size (ESS), and the probability of direction (pd). Parameters include relative condition ($K_{n}$) cumulative vegetation per kangaroo (CVPK), annual juvenile survival (AJS), current reproductive effort (RE), and the random intercept standard deviation (SD).

| **Model I – Subsequent reproduction probability at age 2** | | | | | | | | |
| --- | --- | --- | --- | --- | --- | --- | --- | --- |
| Fixed effects | | | | | | | | |
| Parameter | Estimate | Error | 95% Credible interval | pd | | Rhat | Bulk ESS | Tail ESS |
| Intercept | -0.97 | 0.55 | [-2.08, 0.05] | 97% | | 1.00 | 3110 | 2922 |
| Scaled leg length | 0.77 | 0.24 | [ 0.32, 1.26] | 100% | | 1.00 | 9386 | 7109 |
| Scaled VPK | 0.12 | 0.39 | [-0.58, 0.95] | 61% | | 1.00 | 4384 | 4877 |
| Scaled $K_{n}$ | 0.44 | 0.24 | [-0.02, 0.92] | 97% | | 1.00 | 8399 | 6127 |
| Scaled AJS | 0.71 | 0.34 | [-0.01, 1.47] | 98% | | 1.00 | 5632 | 6576 |
| Random effects | | | | | | | | |
| Parameter | Estimate | Error | 95% Credible interval | | | Rhat | Bulk ESS | Tail ESS |
| Cohort intercept SD | 1.28 | 0.73 | [ 0.14, 3.05] | | | 1.00 | 2184 | 2526 |
| **Model II - Subsequent reproduction probability in ages 3-5** | | | | | | | | |
| Fixed effects | | | | | | | | |
| Parameter | Estimate | Error | 95% Credible interval | pd | | Rhat | Bulk ESS | Tail ESS |
| Intercept | -1.58 | 1.36 | [-4.30, 1.06] | 88% | | 1.00 | 6131 | 7578 |
| Leg | -0.13 | 0.96 | [-2.01, 1.79] | 55% | | 1.00 | 5511 | 5693 |
| Age | 0.93 | 0.38 | [0.22, 1.70] | 100% | | 1.00 | 4952 | 7438 |
| VPK | -1.01 | 1.18 | [-3.38, 1.29] | 80% | | 1.00 | 4574 | 5698 |
| Kn | 1.27 | 0.92 | [-0.47, 3.16] | 92% | | 1.00 | 6018 | 6502 |
| Juvenile survival | -0.41 | 1.25 | [-2.88, 1.99] | 63% | | 1.00 | 4384 | 6730 |
| Current RS (1) | 0.61 | 1.56 | [-2.48, 3.67] | 66% | | 1.00 | 5204 | 7144 |
| Age*Leg | 0.16 | 0.23 | [-0.30, 0.63] | 76% | | 1.00 | 5820 | 6601 |
| Age*VPK | 0.20 | 0.30 | [-0.38, 0.79] | 75% | | 1.00 | 4629 | 5857 |
| Age*Kn | -0.28 | 0.22 | [-0.75, 0.15] | 90% | | 1.00 | 6237 | 6300 |
| Age* Juvenile survival | 0.25 | 0.31 | [-0.32, 0.87] | 80% | | 1.00 | 4698 | 6907 |
| Age* Current RS1 | -0.76 | 0.39 | [-1.51, 0.00] | 98% | | 1.00 | 4274 | 6492 |
| Random effects | | | | | | | | |
| Parameter | Estimate | Error | 95% Credible interval | | | Rhat | Bulk ESS | Tail ESS |
| ID intercept SD | 1.83 | 0.54 | [0.82, 2.99] | | | 1.00 | 1818 | 3095 |
| Cohort intercept SD | 0.72 | 0.44 | [ 0.05, 1.74] | | | 1.00 | 2193 | 3095 |
| **Model III – Total early reproduction** | | | | | | | | |
| Fixed effects | | | | | | | | |
| Parameter | Estimate | Error | 95% Credible interval | | pd | Rhat | Bulk ESS | Tail ESS |
| Intercept | 1.52 | 0.18 | [ 1.19, 1.89] | | 100% | 1.00 | 3744 | 2637 |
| Scaled age | 0.29 | 0.1 | [ 0.11, 0.49] | | 100% | 1.00 | 9147 | 4632 |
|  | 0.02 | 0.21 | [-0.38, 0.45] | | 53% | 1.00 | 4533 | 2996 |
| Age 2 scaled leg length | 0.19 | 0.1 | [-0.01, 0.38] | | 97% | 1.00 | 8932 | 6349 |
| Scaled CVPK | 0.36 | 0.14 | [ 0.08, 0.64] | | 99% | 1.00 | 4438 | 3680 |
| Age 2 scaled $K_{n}$ | 1.52 | 0.18 | [ 1.19, 1.89] | | 100% | 1.00 | 3744 | 2637 |
| Scaled AJS | 0.29 | 0.1 | [ 0.11, 0.49] | | 100% | 1.00 | 9147 | 4632 |
| Random Effects | | | | | | | | |
| Parameter | Estimate | Error | 95% Credible interval | | | Rhat | Bulk ESS | Tail ESS |
| Cohort intercept SD | 0.27 | 0.19 | [0.01, 0.73] | | | 1.00 | 2114 | 2781 |

Supplementary Table 5. Posterior summary of the effects of early reproduction models with the Gelman-Rubin statistic (Rhat), bulk and tail effective sample size (ESS), and the probability of direction (pd). Early primiparity model uses “no early primiparity” as reference for the age 3 primiparity fixed effect. Parameters include early-life reproduction (ER), relative body condition ($K_{n}$), cumulative vegetation per kangaroo (CVPK), vegetation per kangaroo (VPK), metabolic scaling parameter (MSP), environmental parameter (EP), the environmental-behavioural interaction parameter (EBIP), and intercept standard deviation (SD).

| **Model IV - Early- on late-life reproduction model** | | | | | | | | | |
| --- | --- | --- | --- | --- | --- | --- | --- | --- | --- |
| Fixed effects | | | | | | | | | |
| Parameter | | Estimate | Error | | 95% Credible interval | pd | Rhat | Bulk ESS | Tail ESS |
| Intercept | | 0.59 | 0.38 | | [-0.15, 1.35] | 95% | 1.00 | 4019 | 2846 |
| Scaled age | | 0.34 | 0.22 | | [-0.10, 0.78] | 94% | 1.00 | 12195 | 6437 |
| Scaled total reproduction in ages 3-5 | | 0.55 | 0.26 | | [ 0.06, 1.07] | 99% | 1.00 | 8352 | 6223 |
| Age 5 scaled leg length | | 0.12 | 0.24 | | [-0.33, 0.59] | 69% | 1.00 | 9761 | 7144 |
| Scaled CVPK between ages 1-5 | | 0.24 | 0.35 | | [-0.46, 0.93] | 75% | 1.00 | 6120 | 4548 |
| Scaled mean AJS between ages 1-5 | | 0.23 | 0.36 | | [-0.49, 0.95] | 74% | 1.00 | 7307 | 6587 |
| Age 5 scaled $K_{n}$ | | 0.03 | 0.24 | | [-0.43, 0.50] | 54% | 1.00 | 9074 | 6997 |
| Random effects | | | | | | | | | |
| Parameter | | Estimate | Error | | 95% Credible interval | | Rhat | Bulk ESS | Tail ESS |
| Cohort intercept SD | | 0.48 | 0.42 | | [0.02, 1.55] | | 1.00 | 3756 | 4133 |
| ID intercept SD | | 0.40 | 0.32 | | [0.0, 1.16] | | 1.00 | 3279 | 3565 |
| *Model V - Early primiparity model on late-life* | | | | | | | | | |
| Fixed effects | | | | | | | | | |
| Parameter | | Estimate | Error | | 95% Credible interval | pd | Rhat | Bulk ESS | Tail ESS |
| Intercept | | 0.33 | 0.37 | | [-0.40, 1.08] | 84% | 1.00 | 5131 | 4391 |
|  | | 0.37 | 0.21 | | [-0.04, 0.77] | 96% | 1.00 | 10689 | 6756 |
| Age 3 primiparity - Yes | | 0.73 | 0.37 | | [-0.02, 1.46] | 97% | 1.00 | 9235 | 6403 |
| Age 5 scaled leg length | | 0.1 | 0.21 | | [-0.31, 0.53] | 69% | 1.00 | 9036 | 7360 |
| Age 5 scaled CVPK | | 0.25 | 0.36 | | [-0.46, 0.95] | 76% | 1.00 | 7227 | 6631 |
|  | | 0.28 | 0.36 | | [-0.39, 1.00] | 79% | 1.00 | 6532 | 6858 |
| Age 5 scaled $K_{n}$ | | 0.09 | 0.23 | | [-0.35, 0.53] | 65% | 1.00 | 9549 | 6590 |
| Random effects | | | | | | | | | |
| Parameter | | Estimate | Error | | 95% Credible interval | | Rhat | Bulk ESS | Tail ESS |
| Cohort intercept SD | | 0.48 | 0.42 | | [0.02, 1.55] | | 1.00 | 3756 | 4133 |
| ID intercept SD | | 0.40 | 0.32 | | [0.02, 1.16] | | 1.00 | 3279 | 3565 |
| **Effects of early reproduction II** | | | | | | | | | |
| *Model VI – von Bertalanffy Growth Function* | | | | | | | | | |
| Fixed effects | | | | | | | | | |
| Parameter | Estimate | | Error | 95% Credible interval | | pd | Rhat | Bulk ESS | Tail ESS |
| Log initial size Intercept | 5.52 | | 0.04 | [5.44, 5.60] | | 100% | 1 | 10265 | 13222 |
| Log initial size scaled early reproduction allocation | 0.01 | | 0.02 | [-0.03, 0.05] | | 64% | 1 | 7984 | 11385 |
| Log MSP Intercept | 0.11 | | 0.12 | [-0.13, 0.34] | | 83% | 1 | 24682 | 14942 |
| Log MSP scaled early reproduction allocation | 0.06 | | 0.02 | [0.02, 0.10] | | 100% | 1 | 17232 | 14056 |
| Log EP intercept | 6.25 | | 0.01 | [6.22, 6.27] | | 100% | 1 | 4208 | 7309 |
| Log EP scaled VPK | 0.00 | | 0.00 | [0.00, 0.00] | | 75% | 1 | 22792 | 18237 |
| EBIP intercept | 0.94 | | 0.02 | [0.89, 0.97] | | 100% | 1 | 4138 | 7245 |
| Random effects | | | | | | | | | |
| Parameter | Estimate | | Error | 95% Credible interval | | | Rhat | Bulk ESS | Tail ESS |
| Cohort log initial size intercept SD | 0.10 | | 0.04 | [0.03, 0.20] | | | 1.00 | 4970 | 4800 |
| Cohort log MSP intercept SD | 0.74 | | 0.20 | [0.42, 1.19] | | | 1.00 | 4917 | 8636 |
| Cohort log EBIP intercept SD | 0.01 | | 0.00 | [0.00, 0.02] | | | 1.00 | 3354 | 8031 |
| ID log initial size intercept SD | 0.12 | | 0.01 | [0.10, 0.15] | | | 1.00 | 7133 | 12789 |
| ID log MSP intercept SD | 0.08 | | 0.02 | [0.04, 0.12] | | | 1.00 | 5698 | 7714 |
| ID log EP intercept SD | | 0.02 | 0.00 | [0.02, 0.02] | | | 1.00 | 8254 | 13252 |
|  | |  |  | |  | |  |  |  |

# **Figures**


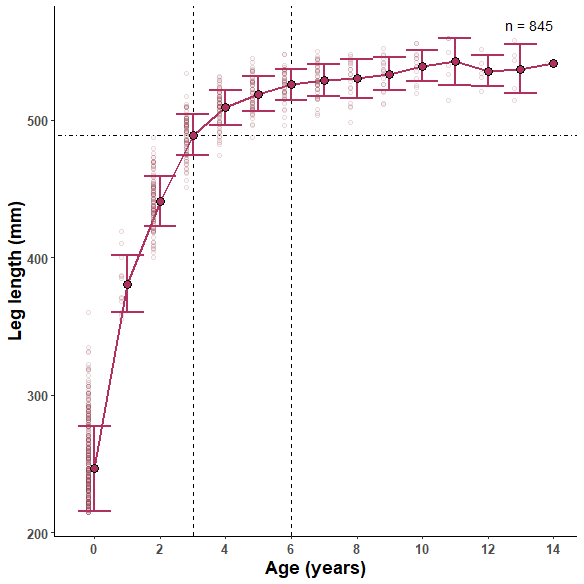


Supplementary Figure 1. Mean leg growth (red line) based on leg measurements taken from female kangaroos (n = 393) of known age, Wilsons Promontory 2010-2023, with mean size (red point) and associated standard deviation (red error bars). Raw measurements (pink points) are displaced left. The horizontal black dashed lines mark ages 3 and 6, with the largest portion of growth post-maturity (age 3). The horizontal dot dash line indicates mean leg length at age 3 of 491mm.


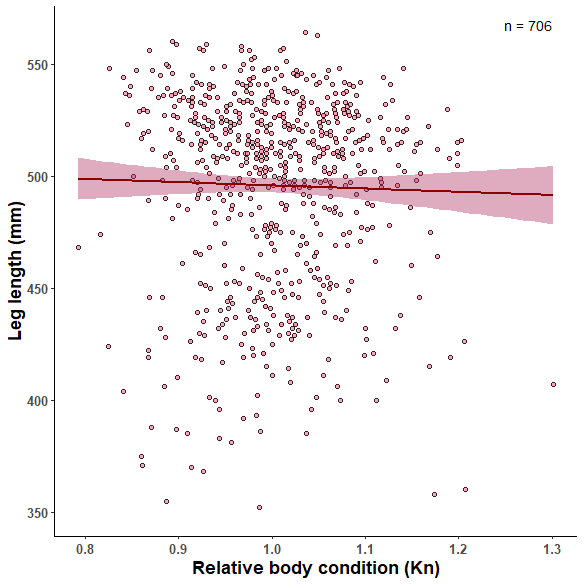


Supplementary Figure 2. The effect of relative condition on the size of female eastern grey kangaroos of known age caught in Wilsons Promontory National Park 2010-2023.


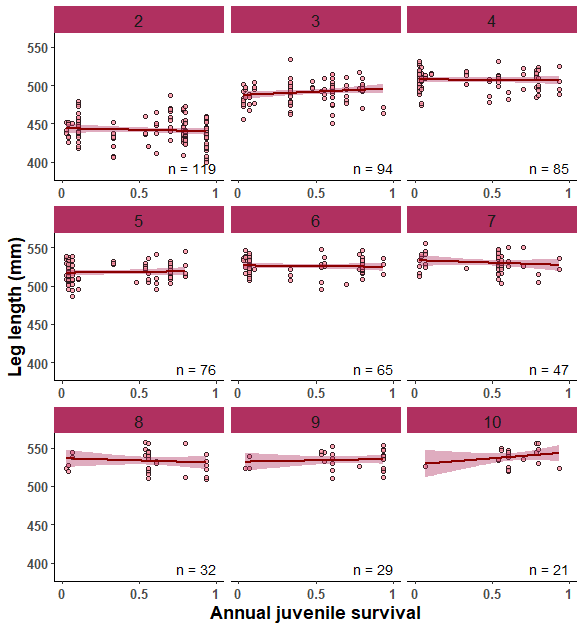


Supplementary Figure 3. The effect of annual juvenile survival on size in female eastern grey kangaroos aged 2-10 years, Wilsons Promontory National Park 2010-2023.


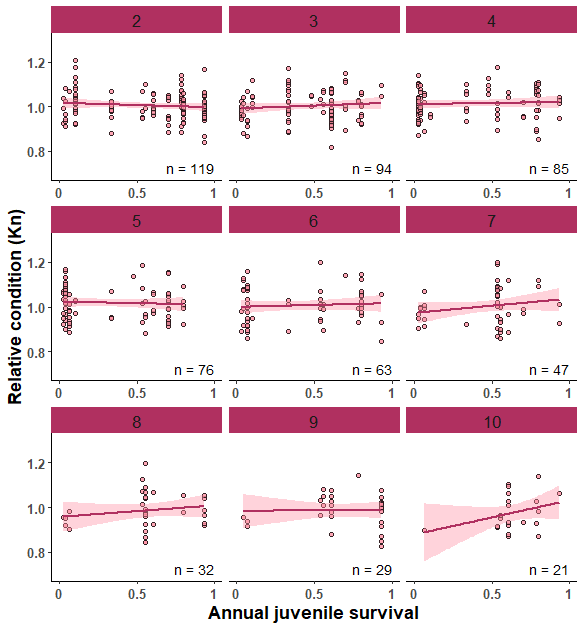


Supplementary Figure 4. The effect of annual juvenile survival on the relative condition in female eastern grey kangaroos aged 2-10 years, Wilsons Promontory National Park 2010-2023.


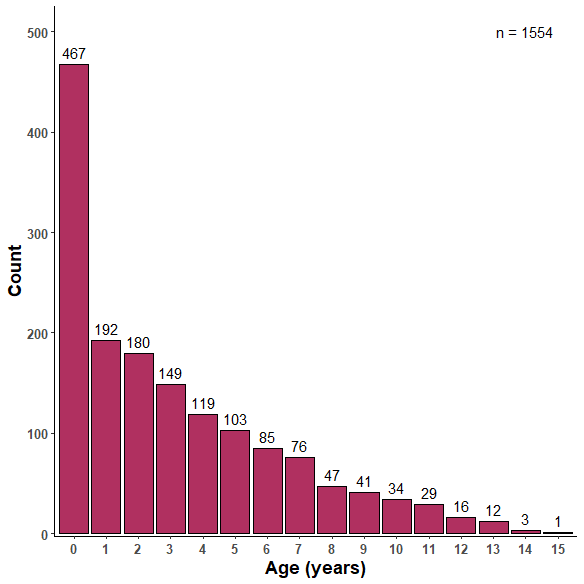


Supplementary Figure 5. Distribution of annual observations per age of 480 known female kangaroos first caught as pouch young until death or to 2023, Wilsons Promontory National Park 2010-2022.


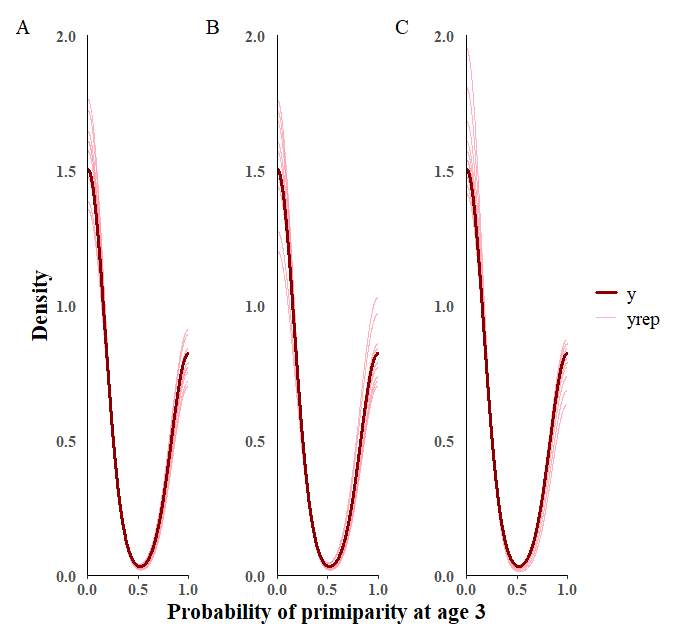


Supplementary Figure 6. Posterior predictive checks to assess Model I’s fit of the response variable, probability of age 3 primiparity, based on the true data (*y*) in red and the predicted or replicated response (*y_rep_*) in pink for non-informative (A), narrow positive (B), and loose positive (C) priors.


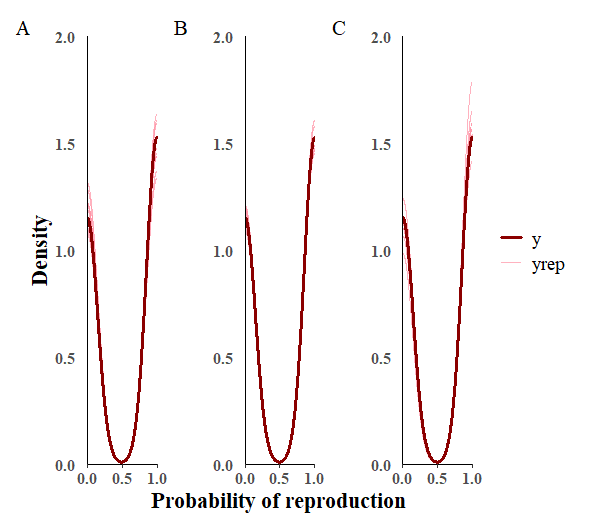


Supplementary Figure 7. Posterior predictive checks to assess Model II’s fit of the response variable, probability of subsequent reproduction in ages 3-5, based on the true data (*y*) in red and the predicted or replicated response (*y_rep_*) in pink for non-informative (A), narrow positive (B), and loose positive (C) priors.


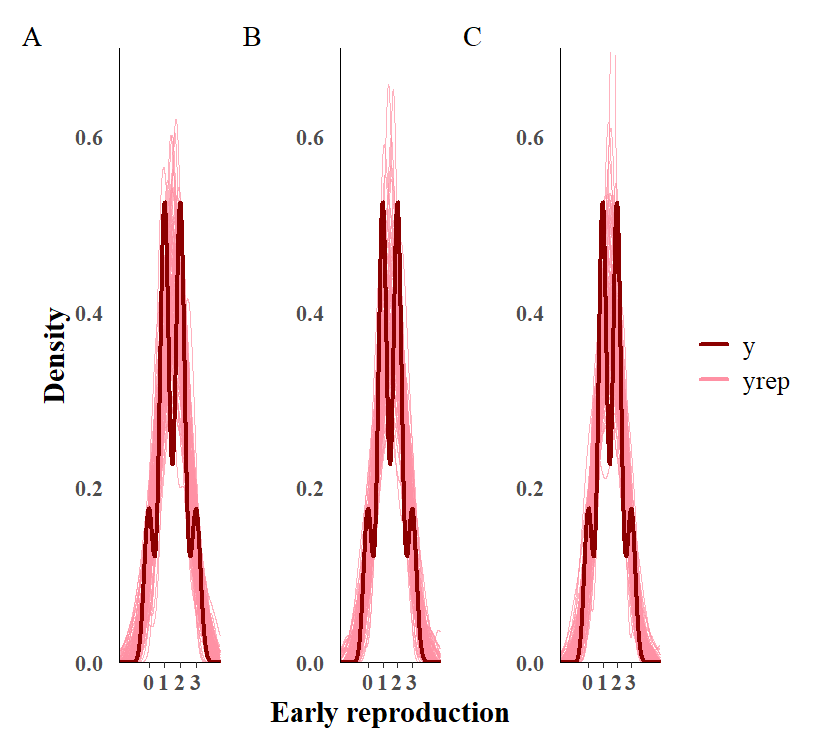


Supplementary Figure 7. Posterior predictive checks to assess Model III fit of the response variable, early reproduction, based on the true data (*y*) in red and the predicted or replicated response (*y_rep_*) in pink for non-informative (A), narrow positive (B), and loose positive (C) priors.


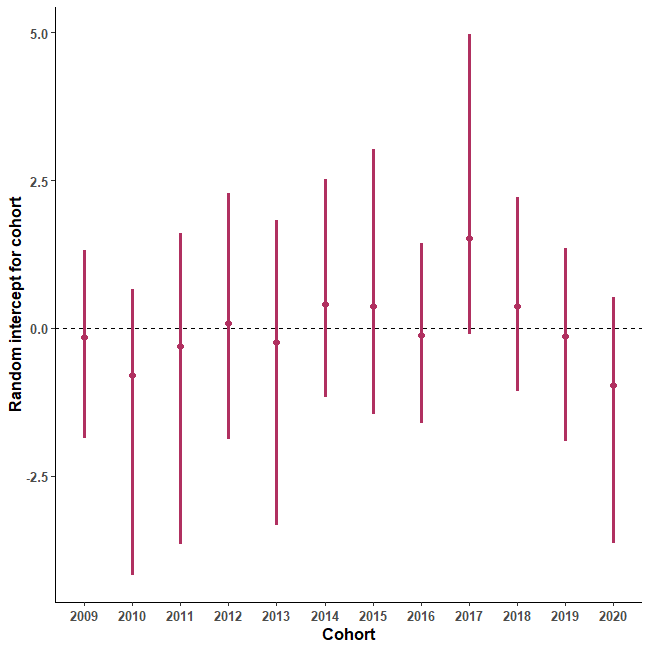


Supplementary Figure 9. The random intercept of cohorts for the probability of primiparity at age 3 with means (red dot) and 95% credible intervals (red lines) in 101 female kangaroos caught between 2009-2022 in Wilsons Promontory National Park.
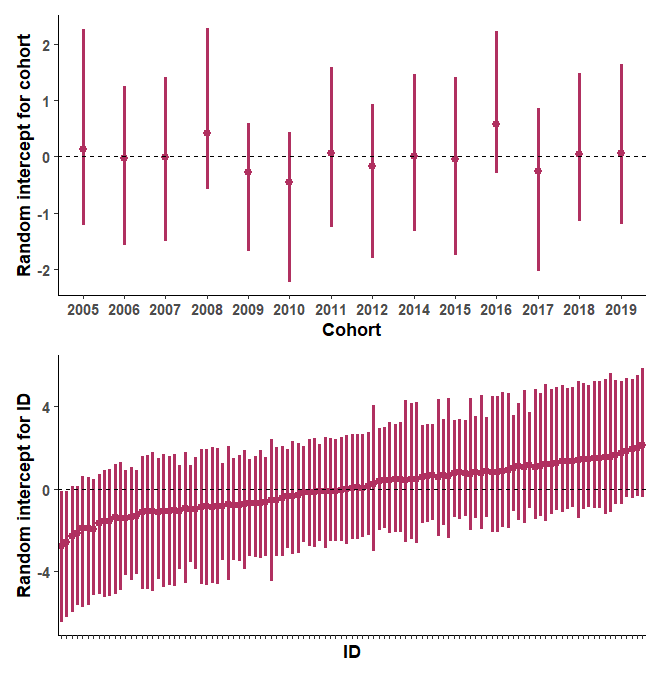


Supplementary Figure 10. The random intercept of cohorts (top) and ID (bottom) for the probability of subsequent reproduction in ages 3-5 with means (red dot) and 95% credible intervals (red lines) in 109 female kangaroos caught between 2009-2022 in Wilsons Promontory National Park.


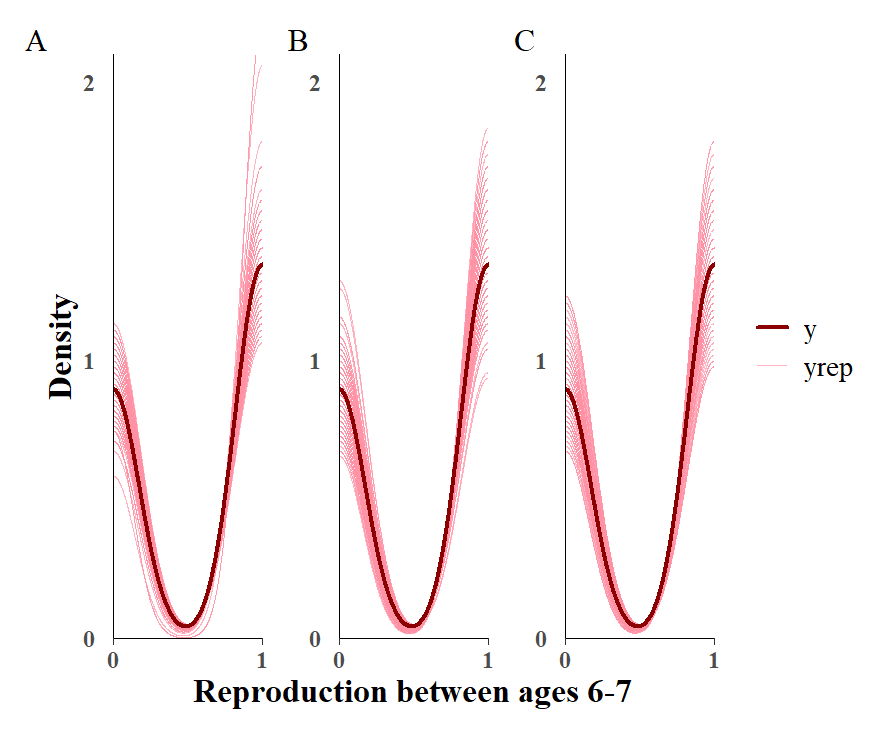


Supplementary Figure 11. Posterior predictive checks to assess Model IV fit of early-life reproduction on the response variable, later life reproduction, based on the true data (*y*) in red and the predicted or replicated response (*y_rep_*) in pink for non-informative (A), narrow positive (B), and narrow negative (C) priors.


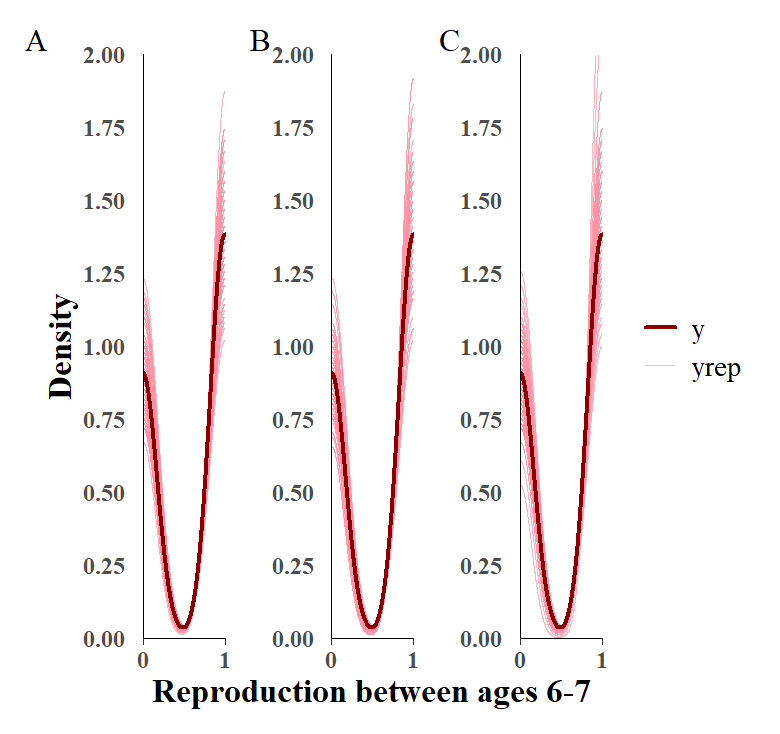


Supplementary Figure 12. Posterior predictive checks to assess Model V fit of early primiparity on the response variable, later life reproduction, based on the true data (*y*) in red and the predicted or replicated response (*y_rep_*) in pink for non-informative (A), narrow positive (B), and narrow negative (C) priors.


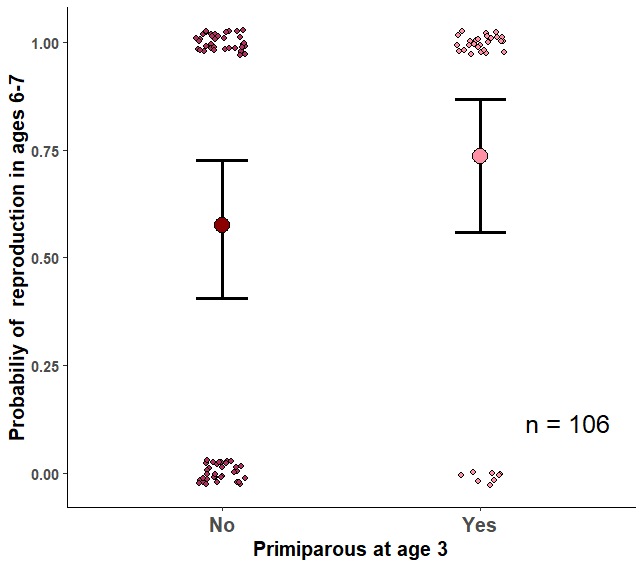


Supplementary Figure 13. Mean conditional predicted effect (coloured large dots) and 95% credible interval (black error bars) of primiparity at ages 3 on the probability of reproductive success in the subsequent year in ages 6-7 of Model V, observed in 106 females between 2010-2018 in Wilsons Promontory National Park. Small coloured points represent raw data.


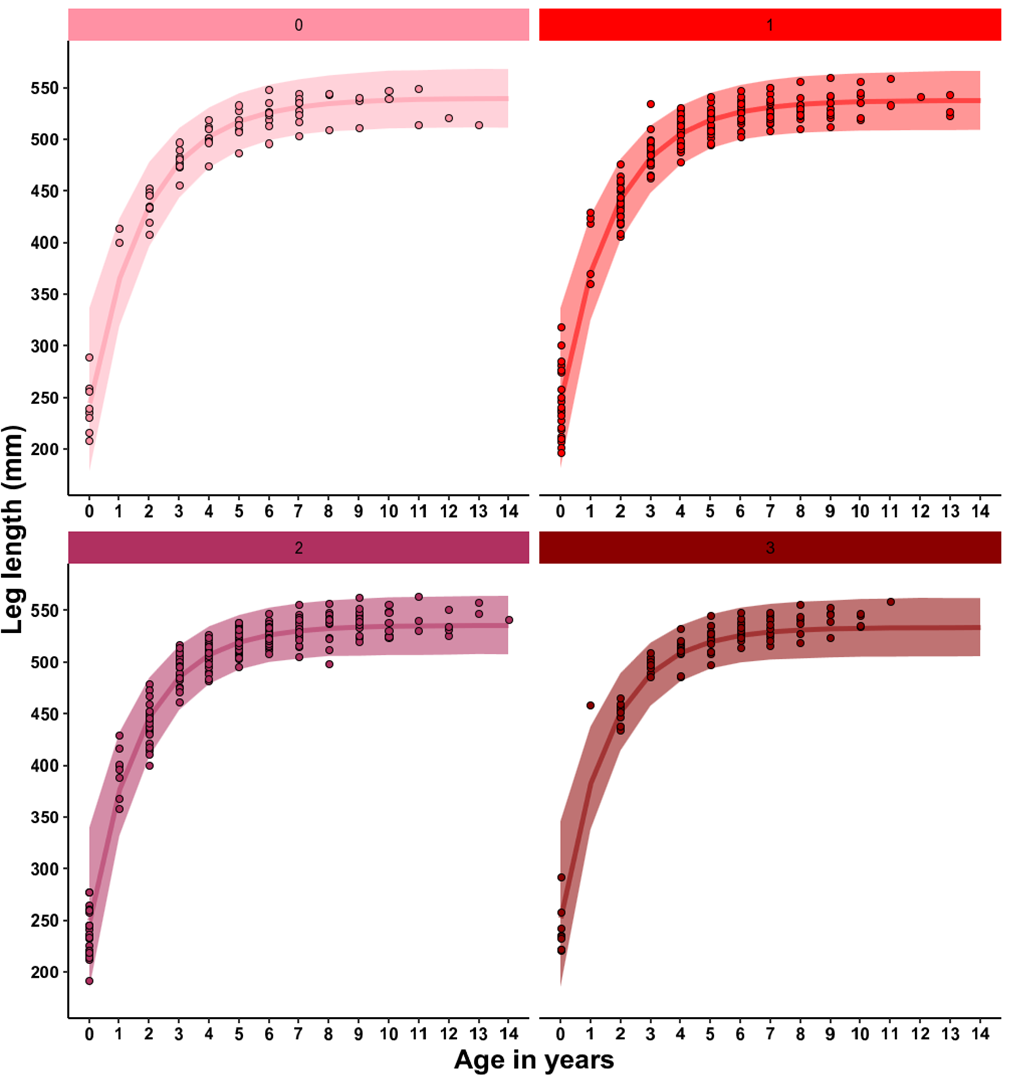


Figure S14. Mean predicted growth curves of female kangaroos according to the number of offspring between ages 3-5. The raw data (dots) include 544 leg measurements for 92 females in 2010-2022, Wilsons Promontory National Park, Victoria, Australia.


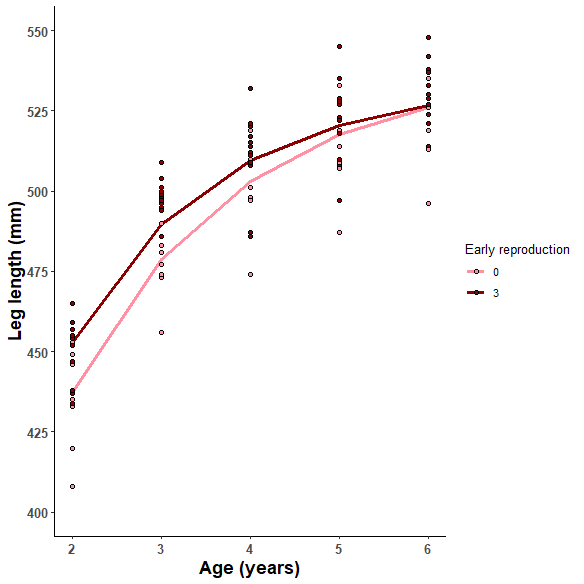


Supplementary Figure 15. Mean predicted growth curve for females that did not reproduce (n = 34 measurements) and those that reproduced at every early reproductive opportunity (n = 51 measurements) between ages 3-5 for 26 female kangaroos caught in Wilsons Promontory National Park between 2010-2022.


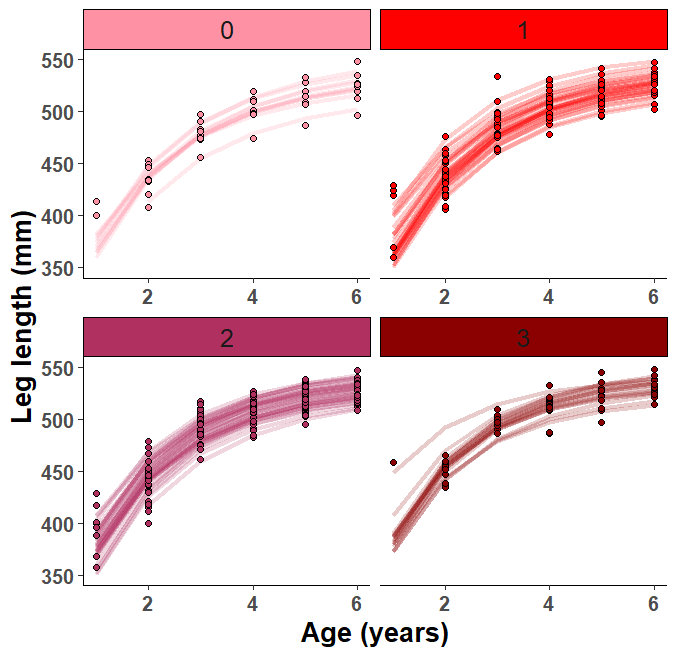


Supplementary Figure 16. Mean predicted growth curves (coloured lines) of known females between ages 1-6, with leg measurements (coloured dots), plotted based on the number of reproductive attempts between ages 3-5.


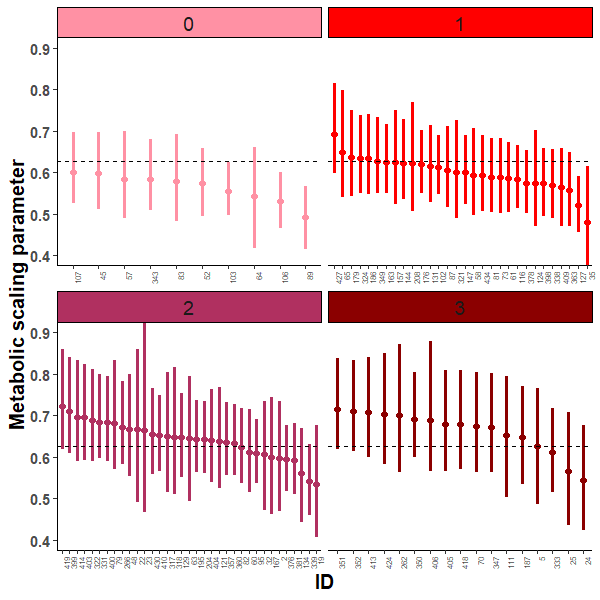


Supplementary Figure 17. Mean predicted metabolic scaling parameter (coloured dots) and 95% credible intervals (coloured lines) of females based on their early reproductive allocation, their ID, and their cohort in (2) of the main article, with a horizontal dashed line representing the mean predicted MSP of the von Bertalanffy growth model. The upper bound for ID 23 exceeds visible range.

.


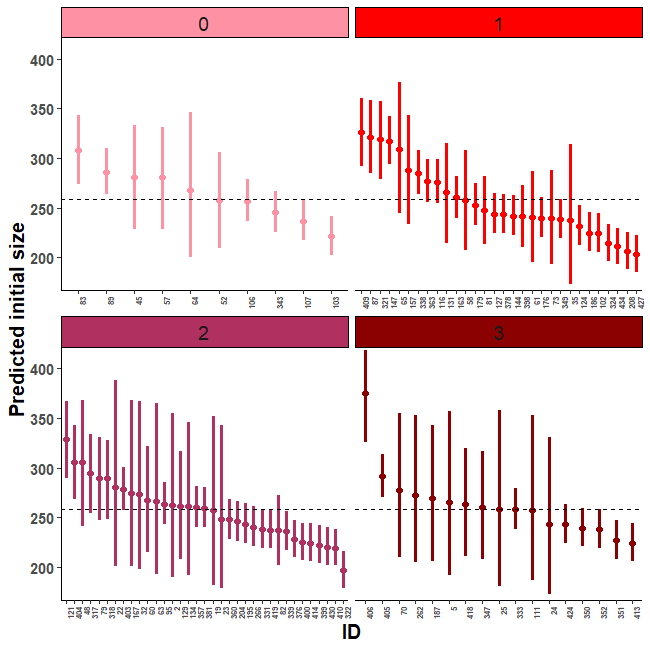


Supplementary Figure 18. Mean predicted initial size as large pouch young (coloured dots) and 95% credible intervals (coloured lines) of females based on their early reproductive allocation, their ID, and their cohort in (2) of the main article, with a horizontal dashed line representing the mean prediction of the von Bertalanffy growth model.


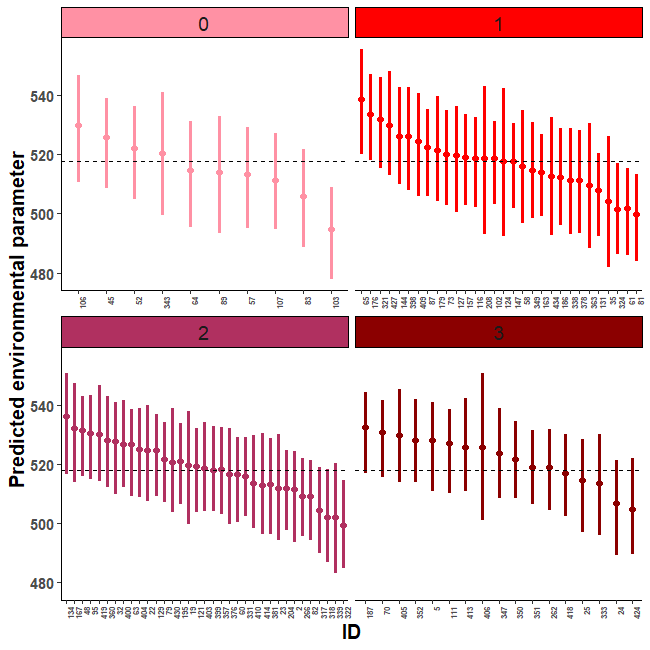


Supplementary Figure 19. Mean predicted environmental parameter (coloured dots) and 95% credible intervals (coloured lines) of females, coloured by their early reproductive allocation, but based on their ID and cohort in (2) of the main article, with a horizontal dashed line representing the mean prediction of the von Bertalanffy growth model.
